# The Place of Industrial Medicine

**Published:** 1920-06-12

**Authors:** 


### THE PLACE OF INDUSTRIAL MEDICINE.

It is only last week that we drew attention to an appeal for a centralised organisation for research into the many problems which industrial medicine presents, and now comes to hand the address recently given by F. Shufflebotham, M.D., M.R.C.P., at the Public Health Conference in Brussels on the "Place of Industrial Medicine in Medical Science."

The lecturer has, it is true, provided no very new conceptions of certain unsatisfactory states which exist in industrial, and particularly colliery life, but in that it is, or should be, realised that the health of the people is one of the principal assets of the nation, then we agree with him that graphic illustrations cannot be too often provided.

Each industry has its special problems and, most large cities having several staple manufactures, it is essential for medical men who practise in such areas to have special knowledge of a number of industrial diseases. It is perhaps unjustifiable to assume that, at present, none of these practitioners possess this facility; because it is common experience that many of them acquire an admirable knowledge of conditions which are never met with and indeed hardly mentioned in the ordinary medical curriculum. The point is, however, that this proficiency has been very slowly acquired, and although there is a very large number of younger men now occupied in gradually gaining this experience on the "assistant" principle, yet to-day the number of practitioners who really understand industrial medicine is, in proportion to the masses under their care, totally inadequate.

Members of the public health services as they stand to-day have opportunity for both collective and individual study of the conditions arising in their particular areas; but by themselves they can achieve relatively little reform. Even were their numbers greatly increased, it is doubtful if much will be accomplished without greatly increased and understanding co-operation from the local medical men. How this can ever arise unless some change is made in the teaching typically provided in our medical schools is a little hard to see.

At present there is practically no provision for the proper teaching of industrial medicine in this country and invariably the young medical man must commence practice with no knowledge of these diseases, and—if one cares so to view the situation—they are largely obliged to obtain their experience at the patients' expense. The obvious result is that thousands of cases may not be diagnosed until too late, and, apart from the then inevitably chronic invalidism imposed upon the patients, many a worker is lost to the country. A thousand examples might be given to show the absolutely urgent necessity for specialised knowledge in industrial practice and yet we have gone on year after year allowing men to enter upon the work with simply the accepted "hospital" training. It is true that in the preliminary training for the various diplomas in public health the problems of industrial disease are very definitely included and also that at the moment a very greatly augmented list of men are taking the trouble to equip themselves with a D.P.H. before commencing even the most general practice, but these very men will be the first to explain that until they decided upon this step, even though they then possessed high medicinal or surgical qualifications, the actual questions associated with industrial medicine had never once, except for one or two stock text-book illustrations, been brought to their notice. All this should change. Not only industrial, but many other branches of professional training are crying out for more attention, and the authorities of our medical schools have much to grapple with. The lecture which lies before us, however, can do much to illustrate these industrial needs and give them a national rather than an academic importance. In many ways they have the first claim to official attention.

